# Supplementary material for: Prior Appendicectomy and Gut Microbiota Re-Establishment in Adults after Bowel Preparation and Colonoscopy
Source: Biomedicines. 2024 Aug 23;12(9):1938. doi: 10.3390/biomedicines12091938 (PMC11429235; doi:10.3390/biomedicines12091938)
Supplement: Supplementary file 1 [file biomedicines-12-01938-s001.zip › biomedicines-2982232-supplementary.pdf]

Supplementary Figure S1. Alpha diversity metrics of **A)** Shannon index and **B)** Richness in those with and without an appendix at baseline. Violin plots show the kernel probability density of the data at different values. Dots represent the mean and the vertical line extends one standard deviation above and below the mean. ns; not statistically significant.

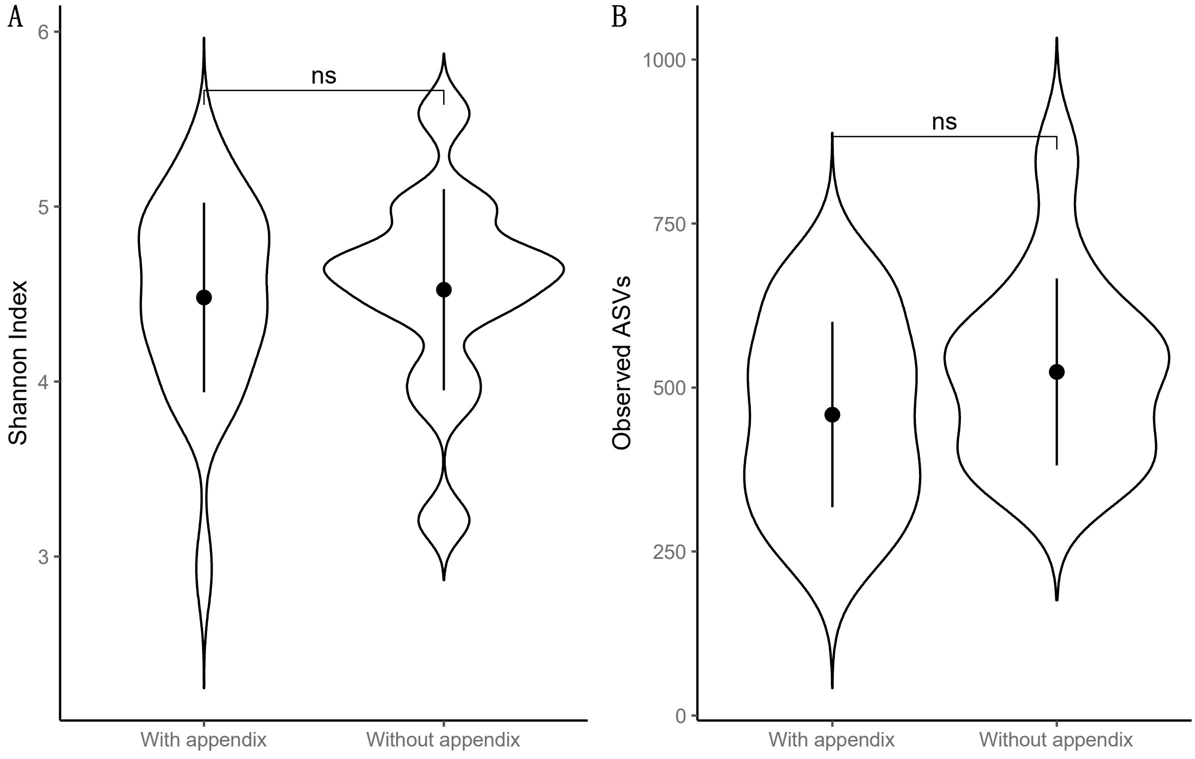

Supplementary Figure S2. Alpha diversity metrics of **A)** Shannon index and **B)** Richness in those with and without an appendix at follow up. Violin plots show the kernel probability density of the data at different values. Dots represent the mean and the vertical line extends one standard deviation above and below the mean. ns; not statistically significant.

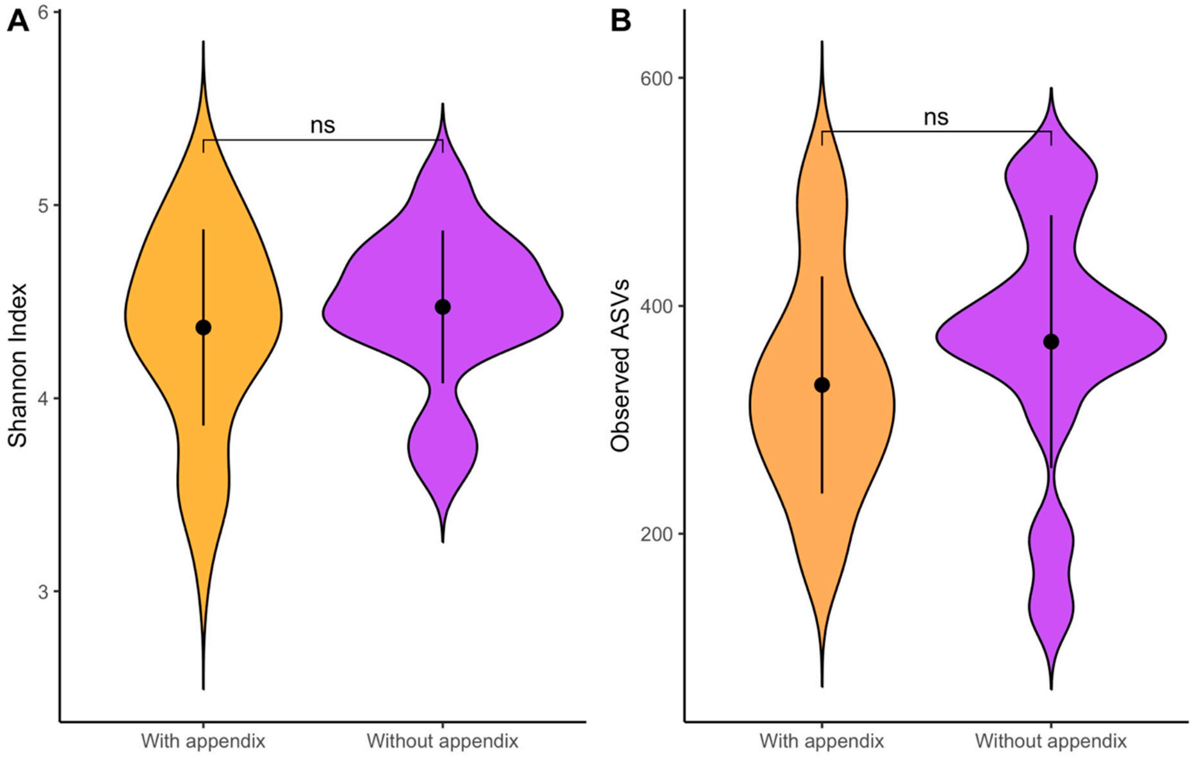

Supplementary Figure S3. Principal components plot based on Aitchison distances (beta diversity) at baseline in those with and without an appendix. Ellipses represent 95% confidence regions according to a fitted multivariate t-distribution.

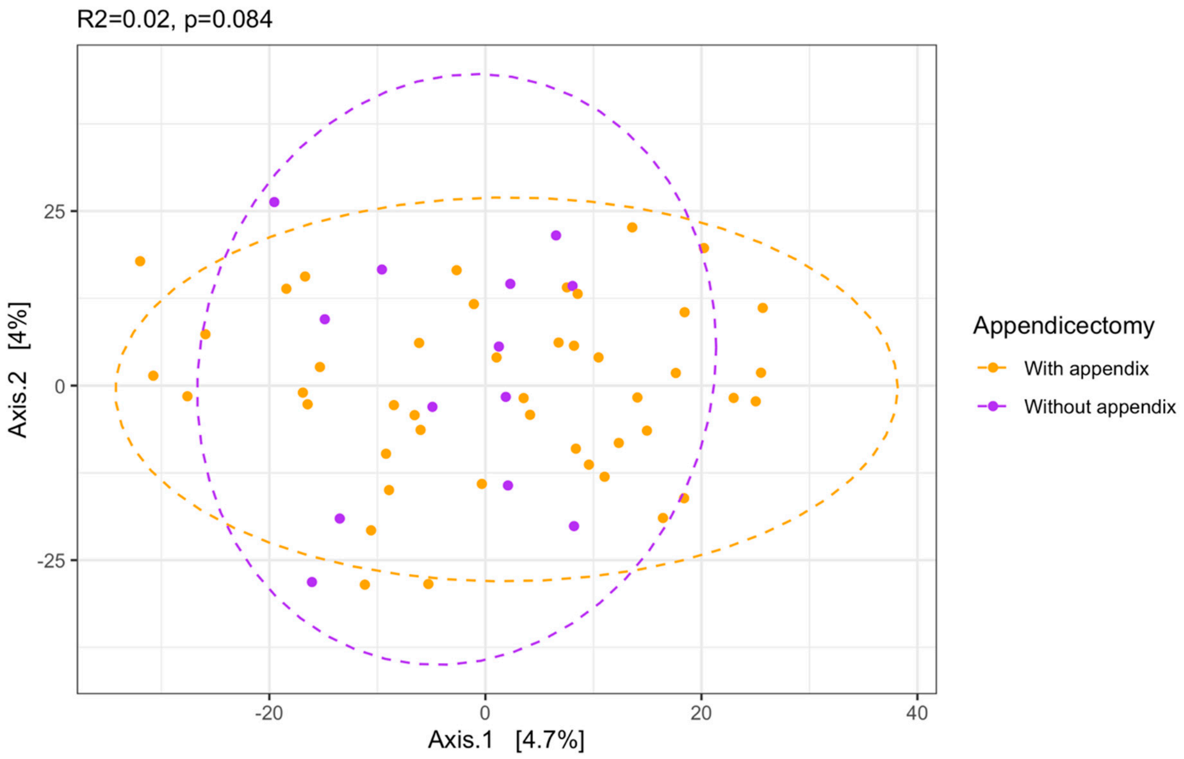

Supplementary Figure S4. Principal components plot based on Aitchison distances (beta diversity) at follow up in those with and without an appendix. Ellipses represent 95% confidence regions according to a fitted multivariate t-distribution.

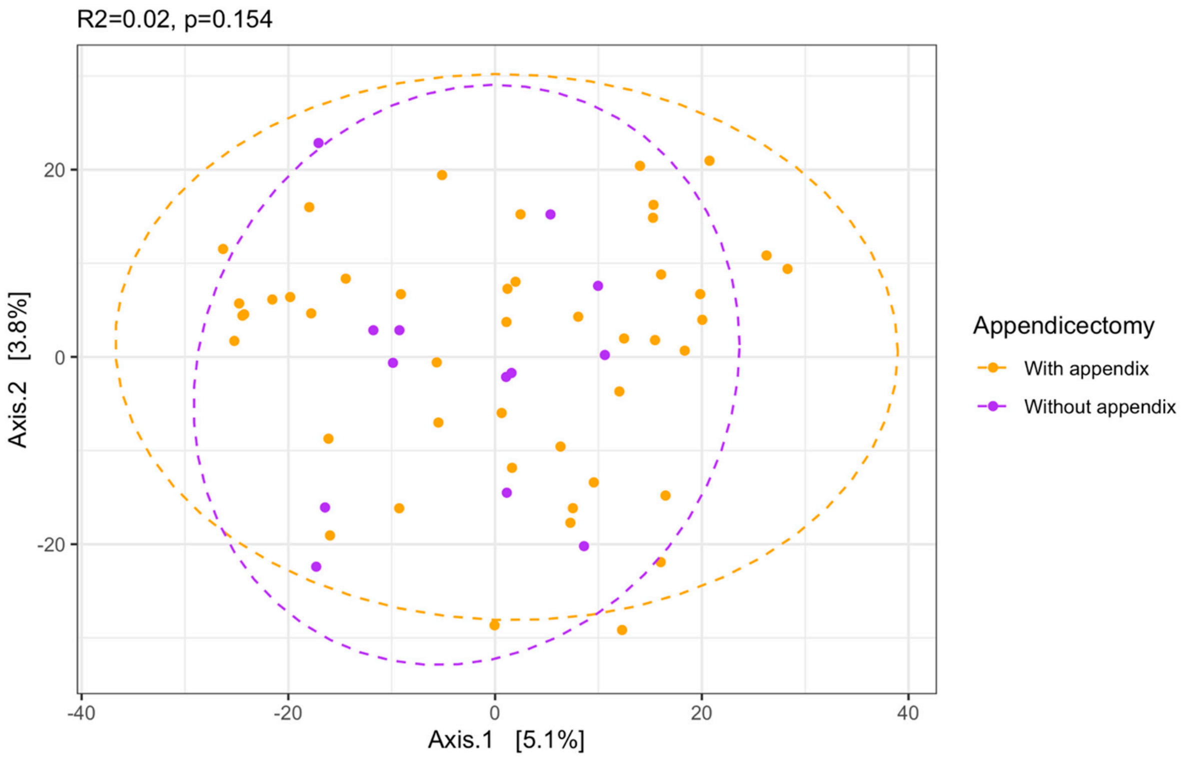

Table S1. Bowel preparation adequacy and colonoscopy indications and outcomes in those with and without an appendix.

	With appendix (n=45)	Without appendix (n=13)
**Sample collection, days**		
One-week pre-intervention, mean (SD)	7.18 (8.11)	4.92 (2.10)
One-month post-intervention, mean (SD)	33.5 (6.94)	33.1 (9.97)
**Bowel preparation adequacy** †		
Excellent	7 (15.6%)	0 (0%)
Good	27 (60.0%)	9 (69.2%)
Fair	11 (24.4%)	4 (30.8%)
**Indication for colonoscopy**		
Positive faecal occult blood test	17 (37.8%)	7 (53.8%)
Surveillance ‡	15 (33.3%)	3 (23.1%)
Other symptom(s) ‡	14 (31.1%)	3 (23.1%)
Change(s) in bowel habit(s) ‡	4 (8.9%)	1 (7.7%)
Iron-deficiency anaemia	4 (8.9%)	1 (7.7%)
Clinically or radiologically detected lesions ‡	1 (2.2%)	1 (7.7%)
**Colonoscopy outcome** §		
Normal	10 (22.2%)	2 (15.4%)
Diverticular disease	14 (31.1%)	4 (30.8%)
Polyp(s)	16 (35.6%)	6 (46.2%)

Notes:

† *Based on the overall Boston Bowel Preparation Scale score (see Supplementary Methods).*

‡ *Surveillance includes for family history, previous polyps, previous gastric surgery; Other symptoms included abdominal pain, peri-rectal bleeding, haemorrhoids, anorectal pain, weight loss, vomiting, bloating, pain on defecation; Change(s) in bowel habit(s) include frequent stools, diarrhoea, faecal incontinence; Clinically or radiologically detected lesions included thickened area on scans and possible rectal mass detected; detected lesions were not associated with a cancer diagnosis.*

§ *Some participants received more than one diagnosis after colonoscopy, therefore percentages exceed 100%.*

¶ *Other outcomes (not tabulated) included haemorrhoids, granular mucosa, and muscle hypertrophy.*

Table S2. Between-group differences in alpha-diversity metrics at baseline and follow up in those with (n=45) and without (n=13) an appendix.

	**Baseline**		**Follow-up**	
Predictors	*Estimate (95% CI)*	*p-value*	*Estimate (95% CI)*	*p-value*
**Shannon index**				
Appendicectomy (Yes)	0.04 (−0.29, 0.38)	0.796	0.11 (−0.15, 0.36)	0.411
**Observed genera**				
Appendicectomy (Yes)	65.0 (−19.9, 150)	0.133	37.9 (−26.2, 102)	0.246

Abbreviations: CI, Confidence interval.

Table S3. Between-group differences in beta-diversity at baseline and follow up in those with (n=45) and without (n=13) an appendix.

	**Aitchison distance**				
Predictors	*Df*	*Sum of squares*	R^2	*F*	*p-value*
**Baseline**					
Appendicectomy	1	5042	0.02	1.10	**0.084**
Residual	56	257036	0.98		
Total	57	262078	1.00		
**Follow up**					
Appendicectomy	1	4661	0.02	1.08	**0.154**
Residual	56	242601	0.98		
Total	57	247262	1.00		

Note: Age at time of recruitment; BMI calculated as weight (kilograms)/height(metres)^2 at time of recruitment; Diet quality measured using a Simple Dietary Questionnaire based on previous studies(37); Depression self-reported at baseline.

Abbreviations: ASVs, amplicon sequencing variants; BMI, body mass index; CI, confidence interval; Df, degrees of freedom.

Table S4. Within-group changes in alpha-diversity metrics after bowel preparation and colonoscopy in those with (n=45) and without (n=13) an appendix.

	**With appendix (n=45)**		**Without appendix (n=13)**	
Predictors	*Estimate (95% CI)*	*p-value*	*Estimate (95% CI)*	*p-value*
**Shannon index**				
Time point	−0.11 (−0.26, 0.04)	0.135	−0.05 (−0.31, 0.21)	0.693
**Observed ASVs**				
Time point	−128 (−166, −89.4)	<0.001	−155 (−220, −89.7)	<0.001

Abbreviations: ASV, amplicon sequencing variant; CI, Confidence interval.

Table S5. Within-group change in beta-diversity after bowel preparation and colonoscopy in those with (n=45) and without (n=13) an appendix.

	Aitchison distance				
Predictors	*Df*	*Sum of squares*	R^2	*F*	*p-value*
**With appendix (n=45)**					
Time point	1	1739	0.004	0.391	**0.047**
Residual	88	391187	0.996		
Total	89	392926	1.000		
**Without appendix (n=13)**					
Time point	1	1418	0.013	0.314	**0.019**
Residual	24	108450	0.987		
Total	25	109869	1.000		
Abbreviations: Df, degrees of freedom.					

Table S6. Within-group changes in genera after bowel preparation and colonoscopy in those with an appendix (n=45), after adjustment for multiple comparisons using Benjamini-Hochberg.

Family	*Genus*	*Coefficient (SE)*	*p-value*	*q-value*
**Lower**				
Lachnospiraceae	Unidentified	−0.37 (0.10)	0.001	0.029
**Higher**				
Uncultured bacterium	Unidentified	0.21 (0.03)	<0.001	<0.001
Propionibacteriaceae	Cutibacterium	0.31 (0.05)	<0.001	<0.001
Veillonellaceae	Megamonas	0.21 (0.04)	<0.001	<0.001
Flavobacteriaceae	Uncultured	0.20 (0.04)	<0.001	0.001
Coriobacteriales Incertae Sedis	Uncultured	1.12 (0.25)	<0.001	0.002
Christensenellaceae	Unidentified	0.18 (0.04)	<0.001	0.004
Ruminococcaceae	Ruminococcaceae UCG-009	0.23 (0.05)	<0.001	0.005
Eggerthellaceae	Gordonibacter	0.22 (0.05)	<0.001	0.005
Unidentified	Unidentified	0.21 (0.05)	<0.001	0.005
Veillonellaceae	Megasphaera	0.18 (0.04)	<0.001	0.007
Acinetobacter sp. CAG-196	Unidentified	0.19 (0.05)	<0.001	0.007
Eggerthellaceae	Uncultured	0.18 (0.05)	0.001	0.029
Ruminococcaceae	DTU-089	0.74 (0.21)	0.001	0.029
Lachnospiraceae	Lachnospiraceae UCG-010	0.17 (0.05)	0.001	0.030
Burkholderiaceae	Oxalobacter	0.22 (0.06)	0.001	0.032
Abbreviations: SE, standard error.				

Table S7. Within-group changes in genera after bowel preparation and colonoscopy in those without an appendix (n=13), after adjustment for multiple comparisons using Benjamini-Hochberg.

Family	*Genus*	*Coefficient (SE)*	*p-value*	*q-value*
**Higher**				
Ruminococcaceae	CAG-352	0.68 (0.10)	<0.001	0.004
Flavobacteriaceae	Uncultured	0.62 (0.11)	<0.001	0.008
Lachnospiraceae	NK4B4 group	0.71 (0.13)	<0.001	0.008
Veillonellaceae	Megamonas	0.63 (0.12)	<0.001	0.008
Lachnospiraceae	Tyzzerella	0.48 (0.09)	<0.001	0.009
Uncultured bacterium	Unidentified	0.52 (0.11)	<0.001	0.010
Erysipelotrichaceae	Holdemanella	0.59 (0.14)	0.001	0.028
Family XIII	Mogibacterium	0.58 (0.15)	0.002	0.038
Burkholderiaceae	Oxalobacter	0.48 (0.13)	0.003	0.043
Erysipelotrichaceae	Unidentified	0.57 (0.15)	0.003	0.043
Pasteurellaceae	Unidentified	0.74 (0.20)	0.003	0.043
Abbreviations: SE, standard error.				

Table S8. Within-group changes in genera after bowel preparation and colonoscopy in those without an appendix (n=13), after adjustment for multiple comparisons using Benjamini-Hochberg.

Family	*Genus*	*Coefficient (SE)*	*p-value*
**Higher**			
Coriobacteriaceae	Collinsella	0.34 (0.13)	0.013
Lachnospiraceae	Ruminococcus gauvreauii group	0.38 (0.16)	0.024
Burkholderiaceae	Unidentified	-0.20 (0.09)	0.032
Lachnospiraceae	Lachnospiraceae NK4B4 group	0.22 (0.10)	0.036
Lachnospiraceae	Lactonifactor	0.18 (0.09)	0.043
Abbreviations: SE, standard error.			
